# Anesthetic Management of Right Single-lung Ventilation in a Patient with Anomalous Left Superior Pulmonary Venous Return for Left Pulmonary Lobectomy

**DOI:** 10.7759/cureus.5780

**Published:** 2019-09-27

**Authors:** Yu Zhang, Allan R Escher, Jonathan B Cohen, Jinhong Liu

**Affiliations:** 1 Anesthesiology, Tianjin Cancer Hospital, Tianjin, CHN; 2 Anesthesiology / Pain Medicine, H. Lee Moffitt Cancer Center and Research Institute, Tampa, USA; 3 Anesthesiology, H. Lee Moffitt Cancer Center and Research Institute, Tampa, USA

**Keywords:** papvr, olv, partial anomalous pulmonary venous return, lung cancer, pulmonary surgery, pulmonary blood flow, right-to-left shunt, malignant melanoma, one-lung ventilation, metastatic melanoma

## Abstract

Partial anomalous pulmonary venous return (PAPVR) is a rare congenital anomaly in which one or more of the pulmonary veins are connected to the right atrium or to the systemic venous system. One lung ventilation (OLV) is required for a number of thoracic procedures. When switching to OLV, right-to-left shunt fraction increases, oxygenation is impaired, and hypoxemia may occur. Hypoxemia during OLV may affect the safety of the patient and is a challenge for the anesthesiologist and the surgeon. This case details the intraoperative anesthetic management of an elderly patient with a PAPVR who underwent single-lung ventilation for lung resection surgery.

## Introduction

Partial anomalous pulmonary venous return (PAPVR) is a form of the extracardiac left-to-right shunt. The cause of PAPVR is unknown, and the severity of this condition is dependent on the proportion of shunt to the total systemic circulation. Total anomalous pulmonary venous return (TAPVR) is not compatible with life [1, 2]. One lung ventilation (OLV) is a functional form of extracardiac right-to-left shunt. OLV is required for a number of thoracic procedures. When switching to OLV, right-to-left shunt fraction increases, oxygenation is impaired, and hypoxemia may occur. Hypoxemia during OLV may affect the safety of the patient and is a challenge for the anesthesiologist and the surgeon. We report a case of an elderly patient with a PAPVR who underwent single-lung ventilation for lung resection.

## Case presentation

The patient is a 71-year-old male with a history of malignant melanoma of the right flank at age 50, which was resected by wide local excision. At age 63, the patient developed a 2.4 cm metastatic melanoma: positive for S100 and HMB45; negative for pancytokeratin, cytokeratin 7 (CK7), cytokeratin 20 (CK20), with leukocyte common antigen (LCA) present in a right groin lymph node. One year ago, the patient developed a 1.8 cm right medial forearm melanoma (positive for melanoma antigen and S100 protein; negative for pancytokeratin), Clark’s level V, Breslow thickness 6.0 mm, with microsatellites and tumor in a small adjacent lymph node (LN) as well as in a right axillary LN; treated by wide local excision of the right forearm site. No residual melanoma was identified. Right axillary lymph node dissection showed metastatic melanoma in five of 20 lymph nodes, with the largest metastatic focus being 3.3 cm. There was no extracapsular extension. Surveillance positron emission tomography (PET) and computed tomography (CT) scans revealed an enlarging (now 1.1 cm) left lower lobe lung nodule with standardized uptake value (SUV) of 4.1. Also, it was identified an anomalous left superior pulmonary vein returning to the left brachiocephalic vein with an estimated 10% shunt.

The patient underwent video-assisted thoracoscopic left lower lobe wedge resection and mediastinal lymph node dissection. Operative time was 97 minutes; estimated blood loss was 150 mL. There were no intraoperative or postoperative complications. Pathology revealed a 1.0 cm poorly-differentiated malignant neoplasm, most consistent with metastatic melanoma (positive for HMB45, Melan-A, and S100; negative for CK7, CK20, TTF-1, and Napsin-A), with negative margins and no lymph node involvement.

This patient was diagnosed with PAPVR from an anomalous left superior pulmonary vein returning to the left brachiocephalic vein, which means the presence of an additional fraction of left-to-right (L-R) shunt in the left lung. We were able to maintain his peripheral oxygen saturation (SpO2) at 96-98% on two lung ventilation with a fraction of inspired oxygen (FiO2) at 30-40%. When OLV applied to this patient during the left thoracotomy, his SpO2 dropped to 88-90%, which was subsequently brought up to 92-93% with supplemental oxygen (↑FiO2 to 60%). Hypoxemia did not recur; there was no indication to increase FiO2 for the remainder of the case. The patient tolerated the remaining procedure well and was discharged home on postoperative day three.

## Discussion

Partial anomalous pulmonary venous return (PAPVR, sometimes also called partial anomalous pulmonary venous connection) is a rare congenital anomaly in which one or more of the pulmonary veins are connected to the right atrium or to the systemic venous system. The incidence of PAPVR in the general population is between 0.4-0.7%, and it is usually incidentally detected in adults during thoracic imaging [1-4]. Most commonly, right-sided PAPVR is detected in children and is associated with atrial septal defects, while left-sided PAPVR is most often found in adulthood [5-6]. Left-sided PAPVR most commonly drains into the left brachiocephalic vein, which was the case in our patient [6]. Most adults with PAPVR are asymptomatic unless there is a significant amount of drainage of the pulmonary blood flow (>50%) to the systemic circulation leading to pulmonary hypertension and right heart failure [7-8]. Consequently, the laterality of the PAPVR in relation to the planned lung resection and the amount of tissue removed are of great importance as they both contribute to the degree of left-to-right shunting which will occur. 

In a case report of a patient requiring a right intrapericardial pneumonectomy for stage I lung adenocarcinoma, a previously unsuspected PAPVR was diagnosed on the contralateral side after the patient developed right ventricular heart failure and hypoxia postoperatively [8]. The patient eventually expired. In another case report, a PAPVR was diagnosed on the ipsilateral side during a left lobectomy for adenocarcinoma [6]. The anesthesia team noted during this case that the patient’s oxygenation was better preserved than anticipated during one-lung ventilation (OLV), presumably due to fact that a portion of the operative (non-ventilated) lung did not contribute to the right-to-left shunt induced by OLV.

In cases where the PAPVR and the tumor are located ipsilaterally but in different lobes, the need for correction of the PAPVR in the asymptomatic patient depends largely on the degree of resection. A larger resection of lung tissue with normal vasculature will result in larger increases in shunt fraction through the PAPVR and/or higher pulmonary artery pressures, and correction of the PAPVR at the time of surgery is likely required [6]. In our case, the 10% shunt through the PAPVR made it unlikely that correction would be necessary, and good outcomes have been reported in patients with uncorrected PAPVR undergoing ipsilateral lobectomies [9]. The decrease in oxygen saturation during the initiation of OLV during this case was likely a result of the expected right-to-left intrapulmonary shunt that OLV induces, as it was readily responsive to an increase in FiO2.

Surgical resection of lung tumors in patients with uncorrected PAPVR can be both feasible and safe. In cases where the tumor arises in the same lobe as the anomalous vein, resection and ligation of the vein is curative for both conditions [6, 10]. In cases where the tumor requires a pneumonectomy or major lung resection, and the PAPVR involves the contralateral side, the PAPVC needs to be addressed prior to tumor resection. Even if a relatively minor resection is planned, hypoxemia may develop if the PAPVR involves the contralateral (dependent) side, owing to the fact that the region of the lung involved with the anomalous vein cannot contribute significantly to the oxygenation of the patient. In cases such as ours, when the tumor is on the ipsilateral side as the PAPVR, but in a different lobe, the anesthesiologist needs to be attuned to the degree of shunt through the PAPVR, as well as signs of increased shunt flow (the development of hypoxemia refractory to supplemental oxygen and right heart failure).

Although this patient’s PAPVR was diagnosed during the workup for his cancer, cases are still initially being discovered intraoperatively [6, 11]. 

## Conclusions

With the increasing trend of laparoscopic and robotic approaches to thoracic tumor resections, anomalous venous connections may not be readily apparent. Anesthesiologists should remain vigilant and consider it as a possible differential diagnosis when treating a patient with hypoxemia and hypotension during and immediately following lung resection.
